# Author Correction: Structures of the human leading strand Polε–PCNA holoenzyme

**DOI:** 10.1038/s41467-024-54128-x

**Published:** 2024-11-08

**Authors:** Qing He, Feng Wang, Nina Y. Yao, Michael E. O’Donnell, Huilin Li

**Affiliations:** 1 Department of Structural Biology, Van Andel Institute, Grand Rapids, MI USA; 2 DNA Replication Laboratory and Howard Hughes Medical Institute, The Rockefeller University, New York, NY USA

**Keywords:** Cryoelectron microscopy, Enzyme mechanisms, DNA synthesis

Correction to: *Nature Communications* 10.1038/s41467-024-52257-x, published online 08 September 2024

In the original version of the published article, the Supplementary Information was missing Supplementary Fig. 9. This has now been replaced.

