# Supplementary material for: Structures of the human leading strand Polε–PCNA holoenzyme
Source: Nat Commun. 2024 Sep 8;15:7847. doi: 10.1038/s41467-024-52257-x (PMC11381554; doi:10.1038/s41467-024-52257-x)
Supplement: Supplementary file 1 — Supplementary Information [file 41467_2024_52257_MOESM1_ESM.pdf]

Supplementary information for

Structures of the human leading strand Polε–PCNA holoenzyme

Qing He, Feng Wang, Nina Y. Yao, Michael E. O'Donnell, and Huilin Li

This document contains:

- Nine supplementary figures
- One supplementary table

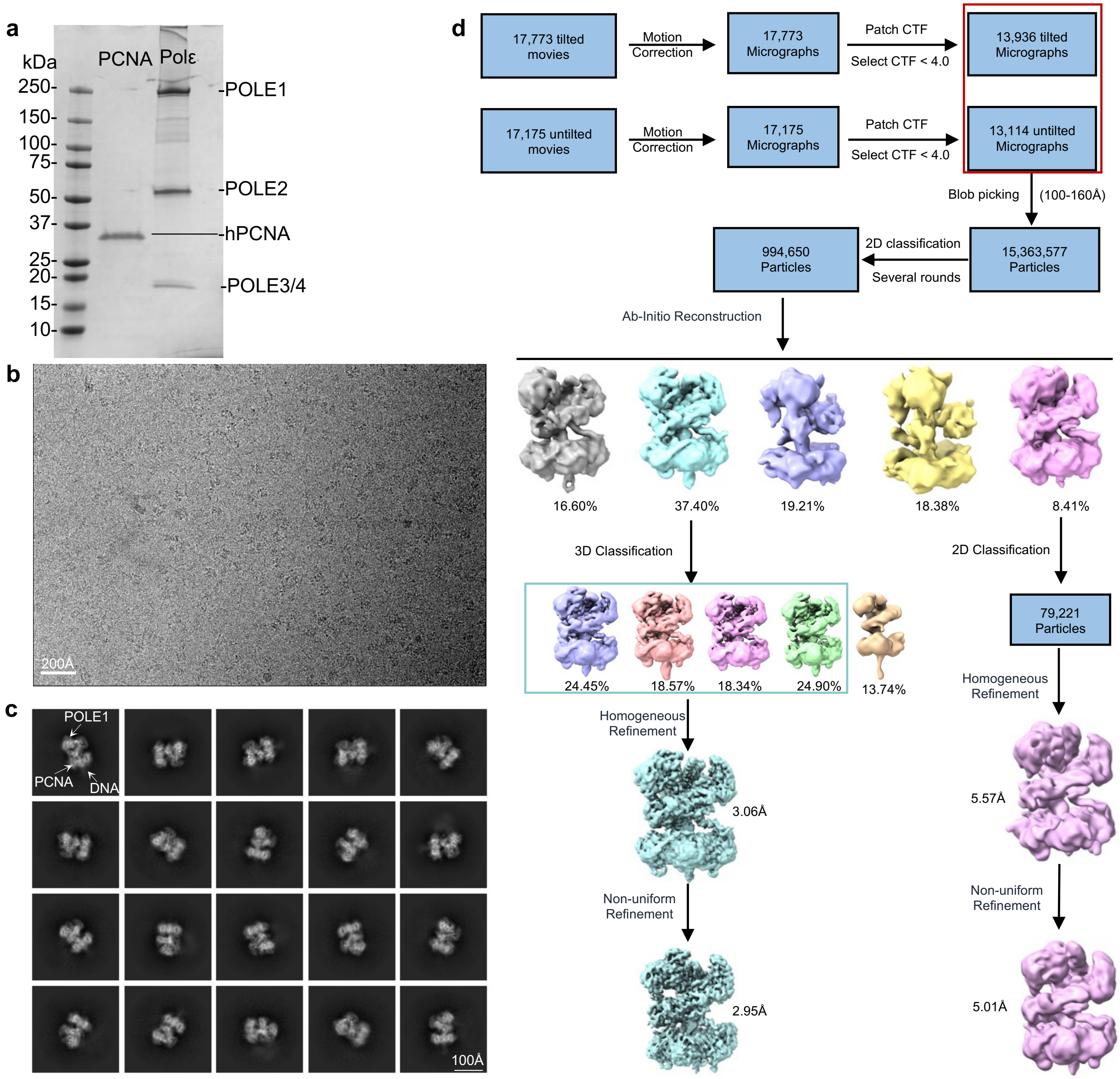

Supplementary Fig. 1. Cryo-EM structure determination of the Polε–PCNA–DNA complex.

a. SDS-PAGE gel of purified human PCNA and Polε holoenzyme. The band for POLE4 is weak due to its small size. Source data are provided with this paper. **b**. A typical raw micrograph of the in vitro assembled Polε–PCNA–DNA ternary complex. A total of 17,175 untilted and 17,773 tilted raw micrographs were recorded. **c**. Selected 2D class averages. **d**. Workflow of cryo-EM data processing and 3D reconstruction in CryoSPARC (version 3.2.0), leading to the two EM maps at 2.95 Å and 5.01 Å respectively.

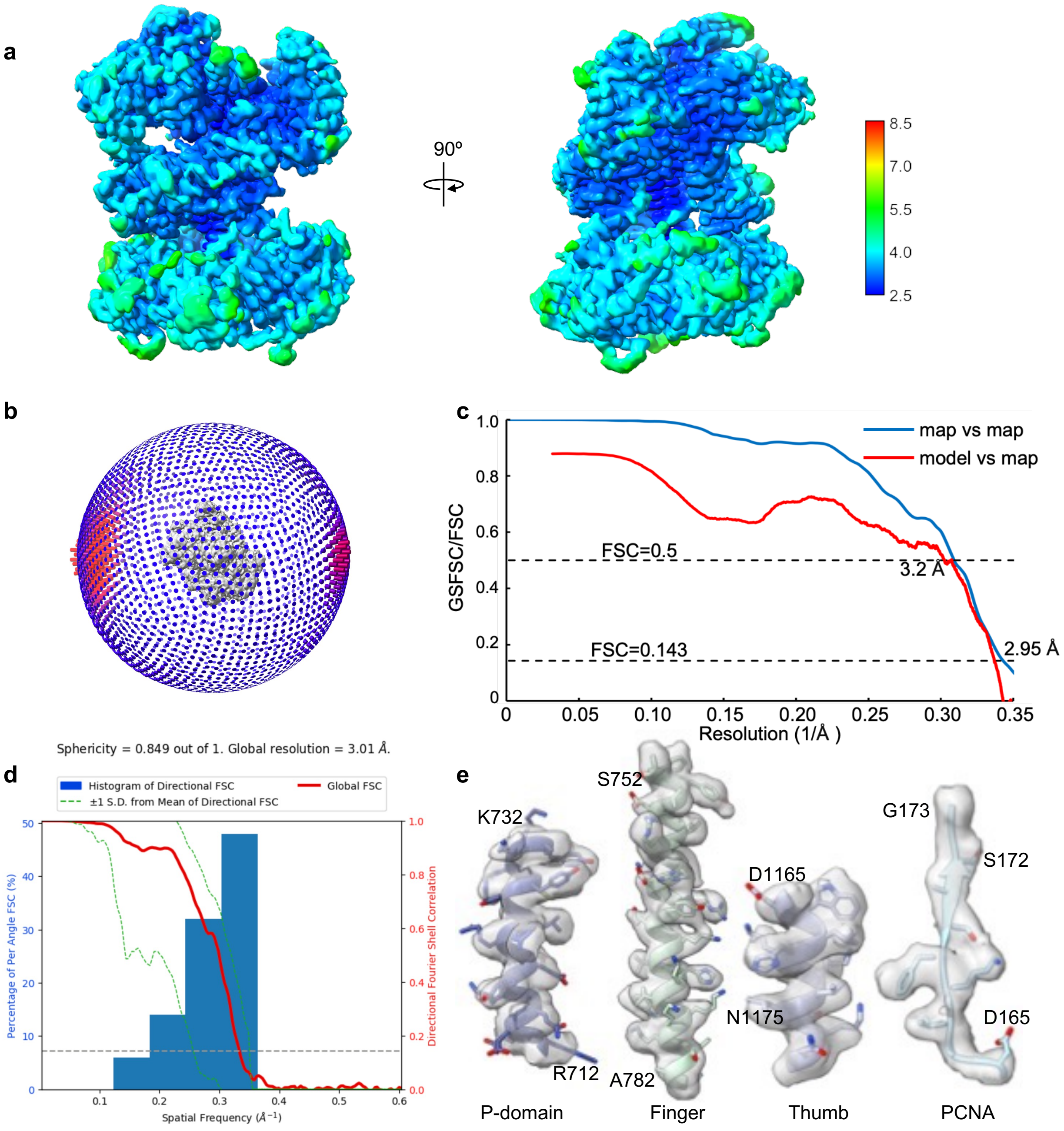

Supplementary Fig. 2. Resolution estimation of the 3D EM map of the Polε–PCNA–DNA complex in the nucleotide bound state. **a**. Color-coded local resolution map in two orthogonal views. **b**. Angular distribution of particle images used in the final 3D reconstruction. **c**. Gold standard Fourier shell correlation (GSFSC) curve of two independent halfmaps (blue) and the Fourier correlation curve of the EM map and the atomic model (red). **d**. Map anisotropy analysis computed by 3DFSC. The grey dotted horizontal line corresponds to FSC = 0.143. **e**. EM densities in four selected regions as labeled.

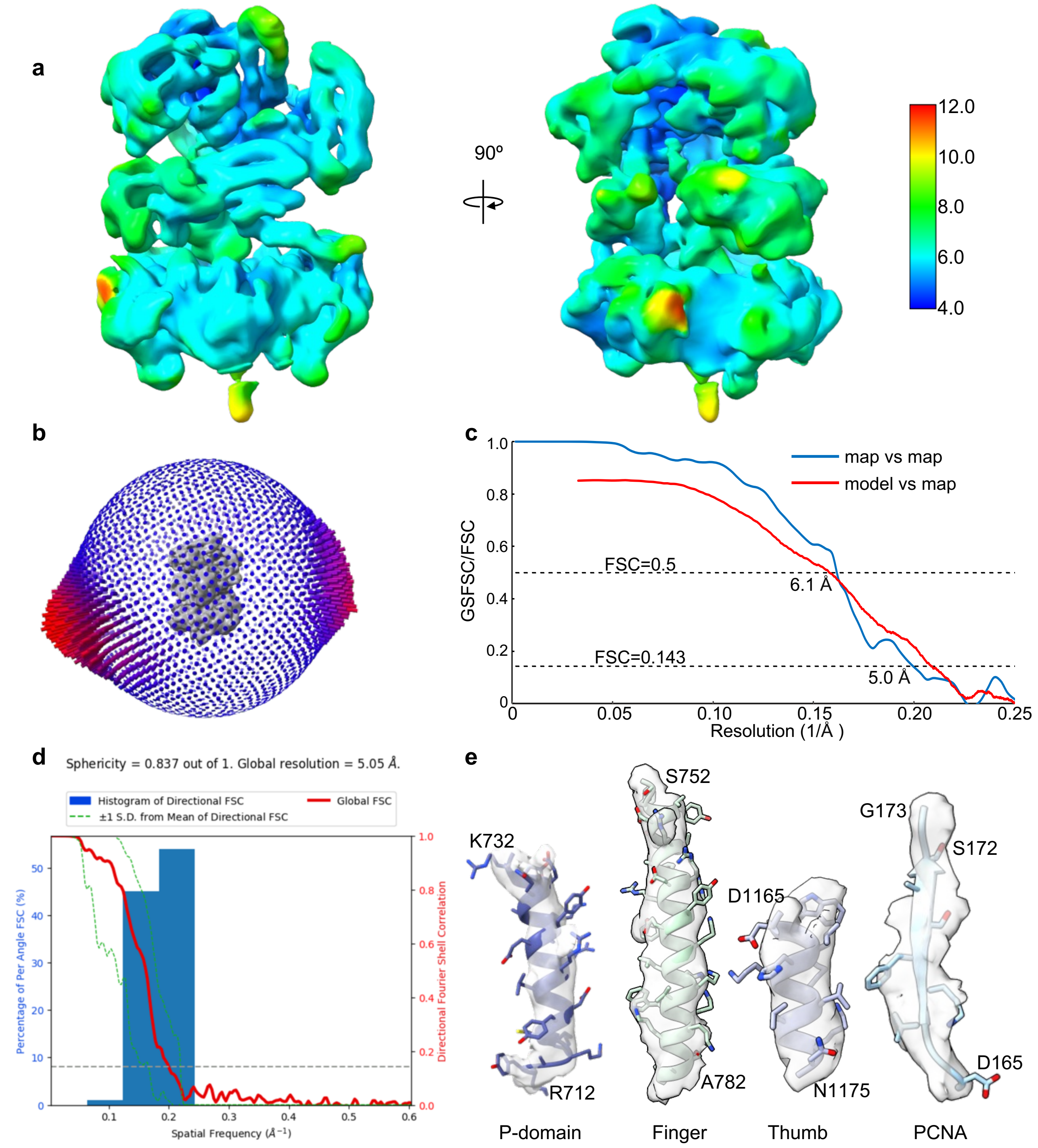

Supplementary Fig. 3. Resolution estimation of the EM map of the Polε–PCNA–DNA complex in the nucleotide exchange state. **a**. Color-coded local resolution map. **b**. Angular distribution of particle images used in the final 3D reconstruction. **c**. Gold standard Fourier shell correlation (GSFSC) of two independent halfmaps (blue) and Fourier correlation curve of the EM map and the atomic model (red). **d**. Map anisotropy analysis computed by 3DFSC. The grey dotted horizontal line corresponds to FSC = 0.143. **e**. EM densities in four selected regions as labeled.

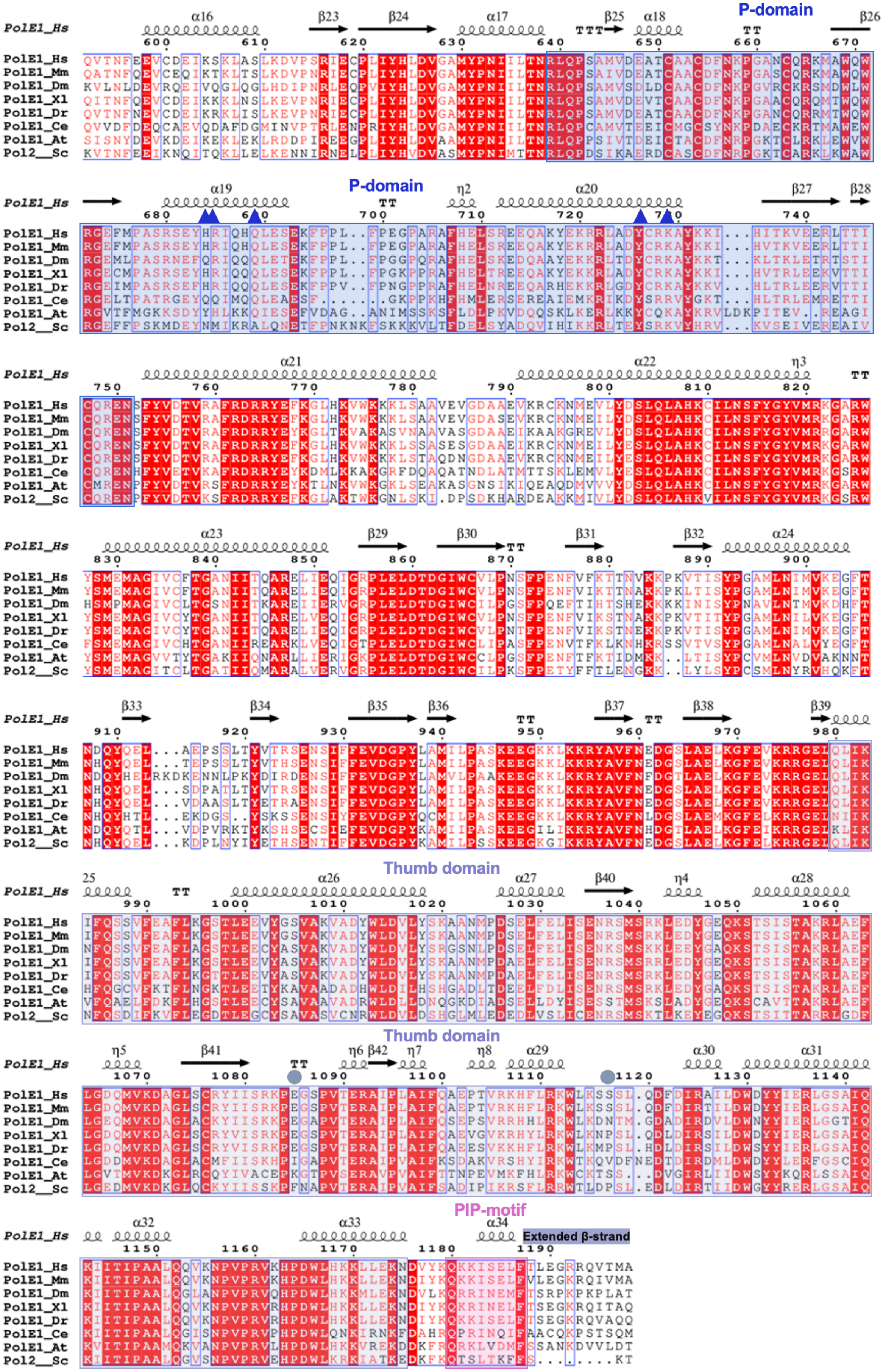
P-domain
P-domain
Thumb domain
Thumb domain
PIP-motif
Extended β-strand
PolE1_Hs
PolE1_Mm
PolE1_Dm
PolE1_Xl
PolE1_Dr
PolE1_Ce
PolE1_At
Pol2__Sc

Supplementary Fig. 4. Sequence alignment of the eukaryotic POLE1-NTD C-terminal half. The blue triangles in the P-domain mark the PCNA-1 binding residues. Three residues in the P-domain of yeast Pol2 are not conserved. The gray circles in the thumb domain mark the residues that weakly bind to PCNA-2. Hs, *Homo sapiens*; Mm, *Mus musculus*; Dm, *Drosophila melanogaster*; Xl, *Xenopus laevis*; Dr, *Danio rerio*; Ce, *Caenorhabditis elegans*; At, *Arabidopsis thaliana*; Sc, *Saccharomyces cerevisiae*.

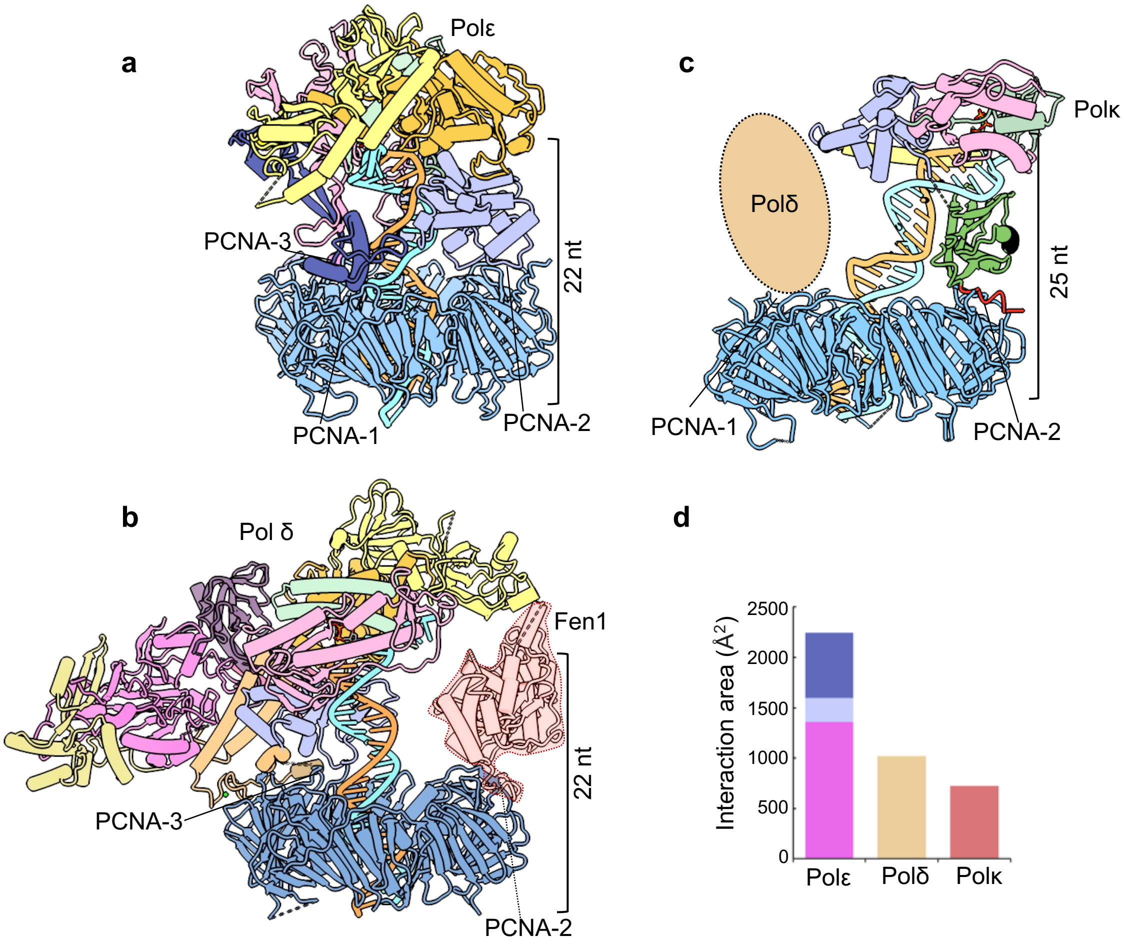

Supplementary Fig. 5. Comparison of three different human polymerase–PCNA–DNA complexes. **a-c**. Cartoon views of the human Polε–PCNA–DNA complex (this study, **a**), the human Polδ–PCNA–DNA complex (PDB ID 6TNY, **b**), and the human Polκ–PCNA–DNA complex (PDB ID 7NV0, **c**). The human PCNA sliding clamp is colored blue, and the catalytic subunit in each complex is colored by domains. The number of nucleotides needed to span the distance from the bottom of PCNA to the activate site of each polymerase is marked. Polδ and Polκ bind to only one PCNA monomer. There is a gap between Polδ and PCNA for Fen1 to bind to another PCNA monomer. There is also a gap between Polκ and PCNA for other PCNA binders. **d**. The interface between the polymerase and PCNA in the three different DNA polymerase-PCNA complexes. Polε binds to all three PCNA monomers and has the largest interface.

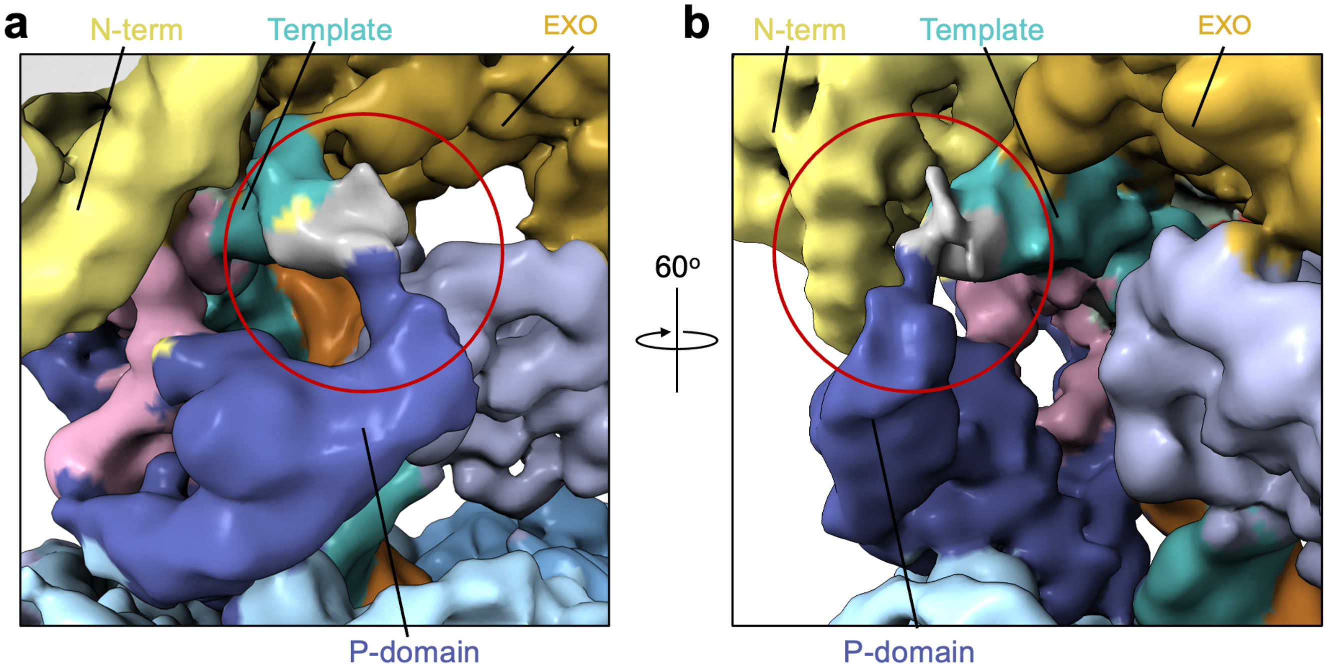

Supplementary Fig. 6. The P-domain contacts the template single strand region in the nucleotide bound state. a-b. Close-up views of the interaction between the P-domain and the template single strand region in the nucleotide bound state. The red circle highlights the connection between the template ssDNA region and the tip of the P-domain. The cryo-EM map is unsharpened and colored by individual domains as in **Fig. 1a**.

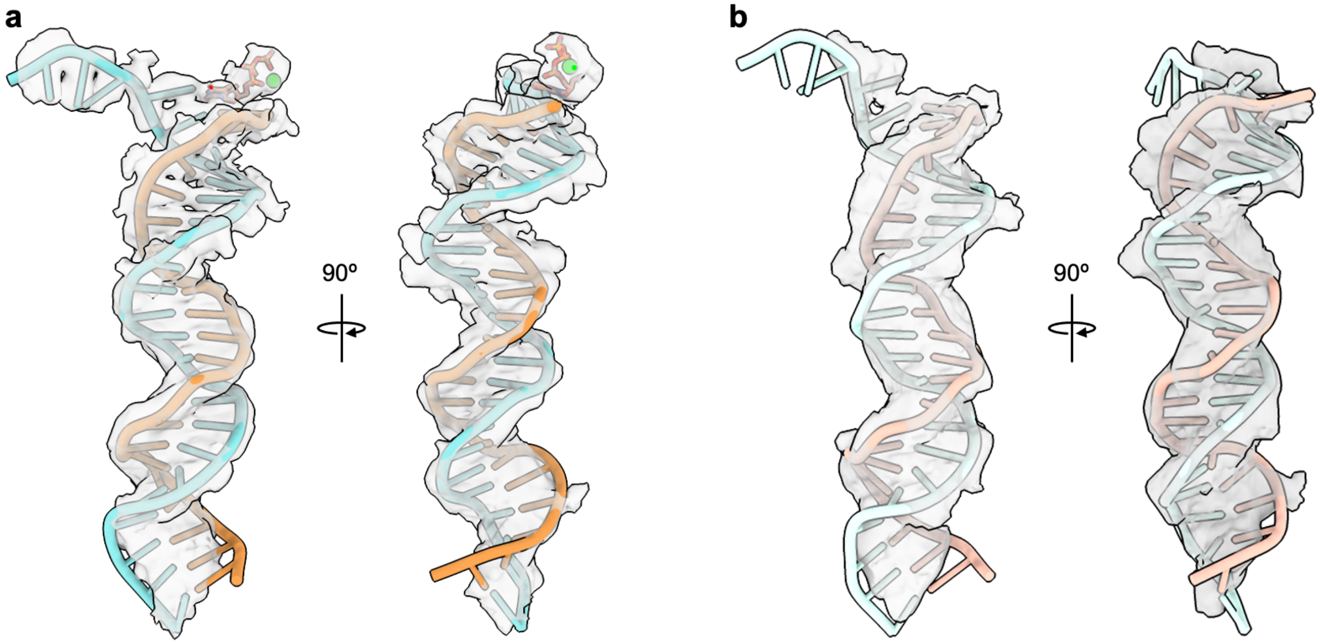

Supplementary Fig. 7. Side-by-side comparison of the P/T DNA structures in the nucleotide bound and the nucleotide exchange states. a. Two orthogonal views of the P/T DNA in the nucleotide bound state superimposed with the EM density rendered in transparent surface. **b**. Two corresponding orthogonal views of the P/T DNA in the nucleotide exchange state superimposed with the EM density rendered in transparent surface. The presence of the incoming dTTP density in (a) and the absence of dTTP at the top of the P/T DNA are evident.

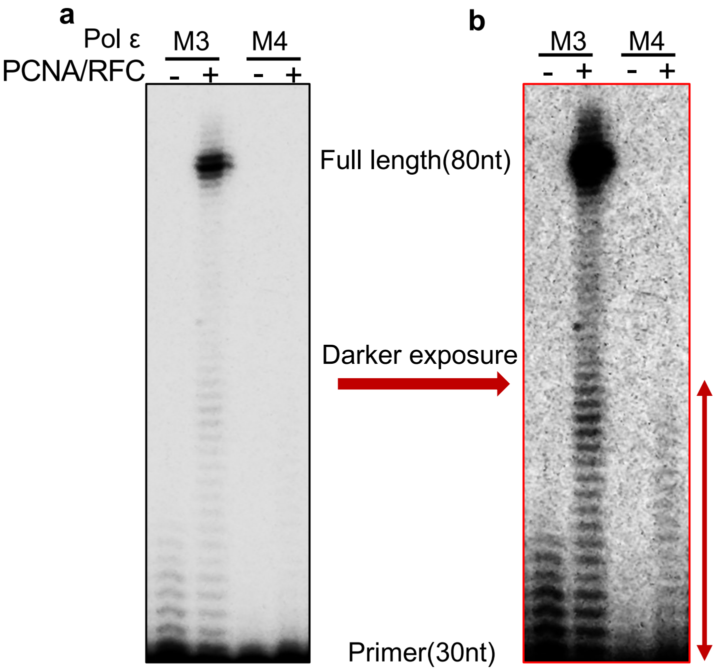

Supplementary Fig. 8. Longer exposure of the primer extension gel image reveals the activity difference of Mut-4 in the presence or absence of PCNA. a. Cropped gel of the Polε P-domain mutants (Mut3-4) with or without PCNA. In this gel image with normal exposure, the difference in Mut-4 activity with or without PCNA is indiscernible. **b**. The same gel region as in **a** but with a longer exposure. The vertical red line marks the primer extension products by Mut-4 in the presence of PCNA. No such short DNA products exit in the absence of PCNA. Source data are provided with this paper.

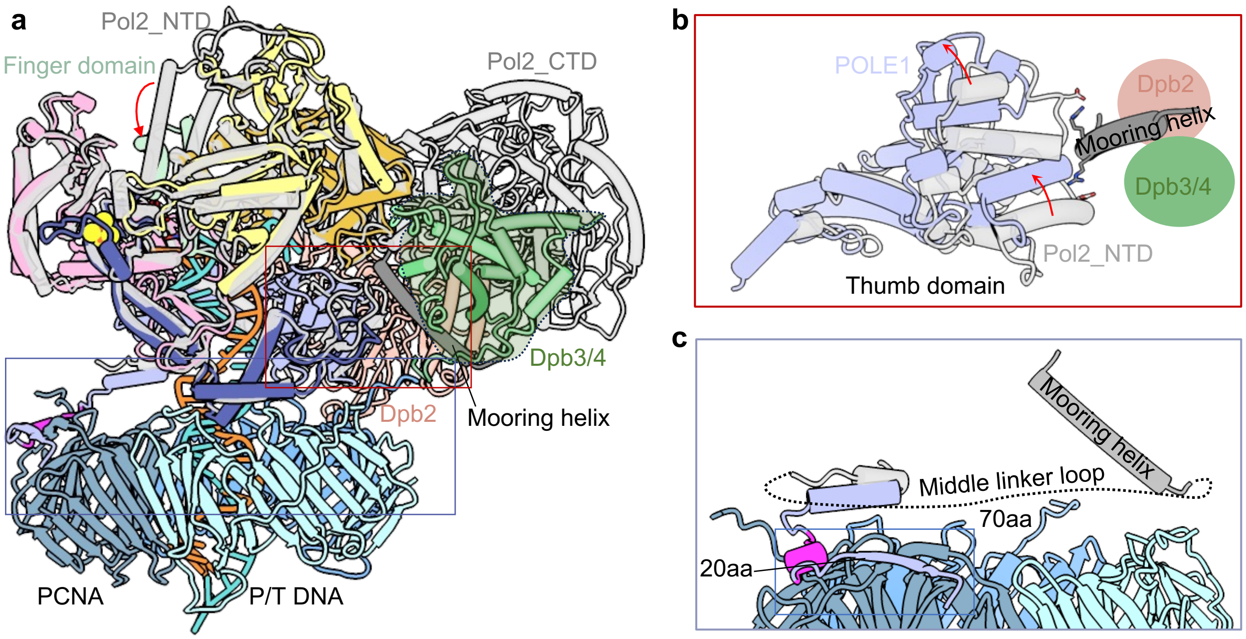

Supplementary Fig. 9. Structural comparison of the human Polε–PCNA–DNA complex and the yeast Polε holoenzyme. a. Superimposition of the human POLE1–PCNA–DNA structure with the yeast Polε holoenzyme structure (PDB ID 6WJV) by aligning the conserved catalytic NTDs. The human POLE1–PCNA–DNA is colored the same as in **Fig. 1c**, the yeast Pol2 is brown with its mooring helix in blue, the yeast Dpb2 is salmon, and Dpb3 and Dpb4 are in light and dark green, respectively. The finger domain is in the "on" and "off" state in the yeast and human complexes, respectively. **b**. Enlarged view of the red boxed region (**a**) showing the mooring helix. The human thumb domain moves away from the mooring helix in the yeast thumb domain, disrupting their interaction. **c**. Enlarged view of the blue boxed region in (**a**) showing the interaction between the PIP motif and PCNA. The middle linker loop (around 70 aa) between the NTD and CTD is flexible in yeast Polε holoenzyme but is partially ordered (the first 20 aa) by interaction with PCNA in the human holoenzyme.

Supplementary Table 1. Cryo-EM data collection, refinement, and validation statistics

	Human Polε–PCNA–DNA complex in the nucleotide bound state (EMD-44358) (PDB 9B8T)	Human Polε–PCNA–DNA complex in the nucleotide exchange state (EMD-44357) (PDB 9B8S)
**Data collection and processing**		
Microscope	FEI Titan Krios	
Magnification	105,000	
Voltage (kV)	300	
Electron exposure (e–/Å^2)	60	
Defocus range (μm)	–1.2 to –1.8	
Pixel size (Å/pixel)	0.828	
Symmetry imposed	C1	
Initial particle images (no.)	994,650	
Final particle images (no.)	320,111	71,210
Map resolution (Å)	2.95	5.01
FSC threshold	0.143	0.143
Map resolution range (Å)	8.5-2.5	12.0-4.0
**Refinement**		
Model resolution (Å)	3.2	6.1
FSC threshold	0.5	0.5
Model resolution range (Å)	30-2.9	35-5.0
Map sharpening *B* factor (Å^2)	-85.1	-244.1
Model composition		
Non-hydrogen atoms	16399	16364
Protein/DNA residues	1929/50	1928/50
Ligands	3	1
B factors (Å^2)		
Protein/DNA	74.25/62.52	218.15/222.85
Ligand	38.44	232.12
R.m.s. deviations		
Bond lengths (Å)	0.005	0.004
Bond angles (°)	0.957	0.927
Validation		
MolProbity score	2.19	1.99
Clashscore	6.98	12.74
Poor rotamers (%)	4.30	0.06
Ramachandran plot		
Favored (%)	95.36	94.63
Allowed (%)	4.64	5.32
Disallowed (%)	0.00	0.05
