# Supplementary material for: Structures of the human leading strand Polε–PCNA holoenzyme
Source: Nat Commun. 2024 Sep 8;15:7847. doi: 10.1038/s41467-024-52257-x (PMC11381554; doi:10.1038/s41467-024-52257-x)
Supplement: Supplementary file 3 — Description of Additional Supplementary Files [file 41467_2024_52257_MOESM3_ESM.pdf]

Description of Additional Supplementary Files

File Name: Supplementary Movie 1
Description: Conformational changes of the human Polε-PCNA-DNA complex from the nucleotide exchange state to the nucleotide bound state. Each domain in Pol ε, PCNA monomer, and DNA are shown in different colours. The overall conformational change was shown first, then the close-up view to show the finger domain pivots around the [4Fe-4S] cluster containing tip of the P-domain to regulate the DNA to bind the incoming nucleotide.
