# Supplementary material for: Structures of the human leading strand Polε–PCNA holoenzyme
Source: Nat Commun. 2024 Sep 8;15:7847. doi: 10.1038/s41467-024-52257-x (PMC11381554; doi:10.1038/s41467-024-52257-x)
Supplement: Supplementary file 5 — Reporting Summary [file 41467_2024_52257_MOESM5_ESM.pdf]

nature portfolio

Corresponding author(s): Huilin Li

Last updated by author(s): 8/23/2024

Reporting Summary

Nature Portfolio wishes to improve the reproducibility of the work that we publish. This form provides structure for consistency and transparency in reporting. For further information on Nature Portfolio policies, see our Editorial Policies and the Editorial Policy Checklist.

Statistics

For all statistical analyses, confirm that the following items are present in the figure legend, table legend, main text, or Methods section.

n/a	Confirmed	
☐	☒	The exact sample size (n) for each experimental group/condition, given as a discrete number and unit of measurement
☐	☒	A statement on whether measurements were taken from distinct samples or whether the same sample was measured repeatedly
☒	☐	The statistical test(s) used AND whether they are one- or two-sided<br>*Only common tests should be described solely by name; describe more complex techniques in the Methods section.*
☒	☐	A description of all covariates tested
☒	☐	A description of any assumptions or corrections, such as tests of normality and adjustment for multiple comparisons
☐	☒	A full description of the statistical parameters including central tendency (e.g. means) or other basic estimates (e.g. regression coefficient) AND variation (e.g. standard deviation) or associated estimates of uncertainty (e.g. confidence intervals)
☒	☐	For null hypothesis testing, the test statistic (e.g. F, t, r) with confidence intervals, effect sizes, degrees of freedom and P value noted<br>*Give P values as exact values whenever suitable.*
☒	☐	For Bayesian analysis, information on the choice of priors and Markov chain Monte Carlo settings
☒	☐	For hierarchical and complex designs, identification of the appropriate level for tests and full reporting of outcomes
☒	☐	Estimates of effect sizes (e.g. Cohen's d, Pearson's r), indicating how they were calculated

Our web collection on statistics for biologists contains articles on many of the points above.

Software and code

Policy information about availability of computer code

Data collection	SerialEM v4.0 (https://bio3d.colorado.edu/SerialEM/)
Data analysis	Relion v4.0 (https://relion.readthedocs.io/en/release-4.0/) and Cryosparc v4.3.0 (https://cryosparc.com). UCSF ChimeraX v1.7.1 for model assembly and docking, AlphaFold-Multimer server, Coot v0.9.8.7 for model building, PHENIX v1.21.1 for model building and refinement, MolProbity v4.5.2 for model validation, deepEMhancer (no version number) for map sharpening.

For manuscripts utilizing custom algorithms or software that are central to the research but not yet described in published literature, software must be made available to editors and reviewers. We strongly encourage code deposition in a community repository (e.g. GitHub). See the Nature Portfolio guidelines for submitting code & software for further information.

Data

Policy information about availability of data

All manuscripts must include a data availability statement. This statement should provide the following information, where applicable:

- Accession codes, unique identifiers, or web links for publicly available datasets
- A description of any restrictions on data availability
- For clinical datasets or third party data, please ensure that the statement adheres to our policy

The two EM maps of the Polε–PCNA–DNA complex have been deposited in the EMDB under accession code EMD-44358 (the nucleotide bound state, 2.95 Å) and

EMD-44357 (the nucleotide exchange state, 5.01 Å). Their corresponding atomic models have been deposited in the Protein Data Bank under accession codes 9B8T and 9B8S.

Research involving human participants, their data, or biological material

Policy information about studies with human participants or human data. See also policy information about sex, gender (identity/presentation), and sexual orientation and race, ethnicity and racism.

Reporting on sex and gender	n/a
Reporting on race, ethnicity, or other socially relevant groupings	n/a
Population characteristics	n/a
Recruitment	n/a
Ethics oversight	n/a

Note that full information on the approval of the study protocol must also be provided in the manuscript.

Field-specific reporting

Please select the one below that is the best fit for your research. If you are not sure, read the appropriate sections before making your selection.

[x] Life sciences   [ ] Behavioural & social sciences   [ ] Ecological, evolutionary & environmental sciences

For a reference copy of the document with all sections, see nature.com/documents/nr-reporting-summary-flat.pdf

Life sciences study design

All studies must disclose on these points even when the disclosure is negative.

Sample size	34,948 movies were collected on Titan Krios, a total of 994,650 particle images were picked and used to reconstitute 3D structures. THe sample size was deemed sufficient when the targeted resolution has been achieved.
Data exclusions	Based on the CTF parameters and the 2D classification, the particles at low resolution or don't generate good averages were excluded.
Replication	Cryo-EM analyses use a large number of particle images in different views. So, there is no need for additional replication
Randomization	Data are not randomized, because they are already randomly distributed when collected off cryo-TEM.
Blinding	Blinding was not relevant to this study

Reporting for specific materials, systems and methods

We require information from authors about some types of materials, experimental systems and methods used in many studies. Here, indicate whether each material, system or method listed is relevant to your study. If you are not sure if a list item applies to your research, read the appropriate section before selecting a response.

Materials & experimental systems

n/a	Involved in the study	
[x]	[ ]	Antibodies
[ ]	[x]	Eukaryotic cell lines
[x]	[ ]	Palaeontology and archaeology
[x]	[ ]	Animals and other organisms
[x]	[ ]	Clinical data
[x]	[ ]	Dual use research of concern
[x]	[ ]	Plants

Methods

n/a	Involved in the study	
[x]	[ ]	ChIP-seq
[x]	[ ]	Flow cytometry
[x]	[ ]	MRI-based neuroimaging

Eukaryotic cell lines

Policy information about cell lines and Sex and Gender in Research

Cell line source(s)	The insect cell line Gibco SF9 (cat. no. 11496015).
Authentication	The commercial cell line from ThermoFisher Scientific (https://www.thermofisher.com/us/en/home/references/gibco-cell-culture-basics/cell-morphology/morphology-of-sf9-cells.html)
Mycoplasma contamination	Negative
Commonly misidentified lines (See ICLAC register)	Not applicable

Plants

Seed stocks	Not applicable
Novel plant genotypes	Not applicable
Authentication	Not applicable
